# Assessment of Single-Word Production for Children under Three Years of Age: Comparison of Children with and without Cleft Palate

**DOI:** 10.1155/2012/724214

**Published:** 2012-04-30

**Authors:** Nancy J. Scherer, Lynn Williams, Carol Stoel-Gammon, Ann Kaiser

**Affiliations:** ^1^Department of Audiology and Speech-Language Pathology, Clinical and Rehabilitative Health Sciences, East Tennessee State University, P.O. Box 70282, Johnson City, TN 37614, USA; ^2^Department of Audiology and Speech-Language Pathology, Center of Excellence in Early Childhood Learning and Development, East Tennessee State University, Johnson City, TN 37614, USA; ^3^Department of Speech and Hearing Sciences, University of Washington, 1417 NE 42nd Street, Seattle, WA 98105, USA; ^4^Department of Special Education, Vanderbilt University, 110 Magnolia Circle, Nashville, TN 37201, USA

## Abstract

*Background*. This study reports comparative phonological assessment results for children with cleft lip and/or palate (CLP) to typically developing peers using an evaluation tool for early phonological skills. *Methods*. Children without clefts (NC = noncleft) and 24 children with CLP, ages of 18–36 months, were evaluated using the *Profile of Early Expressive Phonological* Skills (PEEPSs) [1]. Children interacted with toy manipulatives to elicit a representative sample of target English consonants and syllable structures that are typically acquired by children between 18 and 27 months of age. *Results*. Results revealed significant differences between the two groups with regard to measures of consonant inventory, place of articulation, manner of production, accuracy, and error patterns. Syllable structure did not indicate differences, with the exception of initial consonant clusters. *Conclusions*. findings provide support for PEEPS as a viable option for single-word assessment of children with CLP prior to 3 years of age.

## 1. Introduction

Early intervention based on appropriate and thorough analyses of articulatory and phonological skills is essential; largely due to the role phonology plays in early linguistic development [1–9]. Evidence across a number of studies indicates that phonological abilities, in addition to lexical development, are the two distinguishing characteristics in identifying children as late talkers at an early age [2, 6–8, 10]. For example, Paul and Jennings [7] reported that toddlers who were identified as late talkers had a smaller consonant repertoire and a lower percentage of consonant accuracy compared to typically developing age peers. Based on their findings, Paul and Jennings recommended that the overall number of different consonants in a young child's inventory be used as a sensitive indicator of development and delay and that syllable complexity serves as an effective means for monitoring phonological development. In an investigation of late talker development, Williams and Elbert [9] examined a number of phonetic and phonological measures, including phonetic inventory, syllable structure, syllable diversity, sound variability, percentage of consonants correct, and error patterns. They reported that both quantitative and qualitative phonetic and phonological measures were important in determining recovery from late talking or persistence of a phonological delay.

In addition to phonology's role in early identification, a number of phonetic and phonologic measures have been reported to predict future performance with regard to persistence versus recovery of delayed speech and language development in young children. Carson et al. [2] summarized evidence across several studies that indicate that typically developing children 24–31 months of age had significantly larger and more diverse phonetic inventories for vowels and consonants across all word positions compared to children with SLI-E [10], produced significantly more complex syllables, and were more accurate in their productions than late-talking toddlers [7]. Similarly, Thal et al. [11] reported that children identified as late talkers made greater gains in lexical development if they had larger phonetic inventories and 10 or more words in their vocabularies than children who had smaller phonetic inventories and an expressive lexicon with less than 10 words. Paul and Jennings [7] concluded that global measures of phonological ability, such as syllable structure complexity, phonetic inventory size, and percentage of consonants, can be used as prognostic indicators of development for children identified as late talkers. In a longitudinal study of recovery or persistence of delay, Carson et al. [2] examined 20 different phonological measures, including the number of different consonants (overall and in initial and final positions), number of different consonant clusters (in initial and final positions), and percentage of syllable structures with final consonants. Similar to the quantitative findings reported by Williams and Elbert [9], they found that the greater the delay in phonological development at two years, the more at risk the child was for continuing delays at three years.

Despite the role of phonology in the early identification and prediction of language delay, assessment tools of early phonological skills have not been available [12]. The importance of early phonological assessment is especially important for children with cleft lip and/or palate (CLP) who are at risk for early speech and language delays due to the presence of the cleft during the first year of life [13–18]. For some children, this delay persists through the preschool period and may impact early literacy skills [19–24]. It is thought that early structural deficits restrict the speech sound inventories of young children with CLP, which in turn limits vocabulary growth. It has been established that young children with CLP produce more words beginning with nasals, vowels, glides, and fewer words beginning with oral stop consonants than children without clefts [25]. It is also apparent that they show an early preference for sounds made at the labial, velar, and glottal place of articulation [25].

The recent focus on early intervention for children with CLP attempts to change the trajectory of early speech and language development; however, limitations in speech assessment materials for children under three years restrict the ease and accuracy of speech sound assessment that, in turn, drives goal selection for treatment [18]. Assessment of phonological development for children with CLP, as well as all young children, has been based primarily on parent report (e.g., McArthur Communication Development Inventories) [26] or a brief subtest within a larger standardized test (e.g., *Sequenced Inventory of Communicative Development*; [27]). These measures, however, are extremely limited and cannot provide a representative or even sufficient evaluation of the key phonetic and phonemic characteristics which are required for an age-appropriate clinical assessment. The Parameters of Care from the American Cleft Palate-Craniofacial Association [28] recommend a speech-language evaluation twice in the first two years of life and again at three years of age. A single-word measure of speech production specifically designed to assess the major components of phonological development in young children would be helpful in identifying children who would benefit from early intervention.

Assessment of speech sound development in children less than three years of age is challenging due to the inherent variability in early language and phonological development [29, 30]. This variability is due in part to the strategies young children use to select words from their vocabulary and to the absence of phonological rules that govern the more stable sound system observed in older children [1]. We know that the phonological characteristics of the words play an important role in determining which words a child includes in their early vocabularies for typically developing children, as well as children with cleft lip and palate [25, 31–35]. Children choose words with phonological characteristics that are consistent with their developing sound systems and avoid words with characteristics that are outside their sound system. A second feature of early word learning is that words are produced as an unanalyzed whole. This learning strategy results in significant variability in children's production of speech sounds and poor association between the child and adult model. It is important, therefore, to assess sufficient exemplars with representative phonetic characteristics to check for consistency and variability and to sample words with sounds that do not occur in routine conversation in addition to sounds that the child uses frequently [36, 37].

*The Profiles of Early Expressive Phonological Skills* (PEEPSs; [1]) was developed to assess consonant inventory, place of articulation, manner of production, syllable structure, accuracy, and error patterns of children between 18–36 months of age. PEEPS was constructed to represent the diversity of place, manner, and voicing of English consonant production, as well as different syllable structures. Finally, multiple exemplars of the different phonetic characteristics were included across a number of words in order to check consistency and variability of children's early phonological skills. The purpose of this study was to compare the phonologic development of a group of children with CLP between 18–36 months to a group of children without clefts (NC) using the PEEPS assessment tool.

## 2. Method

### 2.1. Participants

This study was approved by the East Tennessee State University (ETSU) and Vanderbilt University Institutional Review Boards and the study was conducted with the understanding and consent of the parents of the participants. Forty-two children without cleft lip and/or palate and 26 children with cleft lip and/or palate between the ages of 18 and 36 months of age participated in this study. Mean age of the NC group was 25.5 months. (SD = 7.4 months) and mean age of the children with CLP was 27.4 months (SD = 6.5 months). The NC group had 16 males and 26 females and the children with CLP had 16 males and 10 females. No significant gender differences were identified for the phonological components analyzed. The NC children were recruited from the university childcare centers at ETSU. They had no cognitive, speech-language, or hearing impairments, as reported by their parents. The children with cleft lip and palate were part of an ongoing longitudinal study of early intervention at two sites: ETSU in Johnson City, TN, and Vanderbilt University in Nashville, TN. The children with CLP had not received any intervention at the time of this study. All of the children with CLP met the following inclusion criteria: (1) palate repair by 12 months of age; (2) absence of dysmorphology associated with a genetic syndrome according to a geneticist, cognitive delay, or sensorineural hearing loss; (3) monolingual English speaking family. The children with CLP had PE tubes placed bilaterally between the time of lip and/or palate repair. All the children passed a hearing screening at the time of evaluation although 75% of the children had reports of 3–5 middle ear infections since birth. Five children had bilateral CLP, 16 had unilateral CLP, and 5 had cleft palate. The children with CLP had a mean receptive and expressive language standard score of 103 (SD = 7) and 89 (SD = 10), respectively, on the Preschool Language Scale-4 [38].

### 2.2. Assessment Procedures

Each child was assessed individually using the PEEPS [1] either in a clinic setting or in their childcare center by a speech-language pathologist or graduate student in speech-language pathology trained in its administration. The testing took approximately 15–20 minutes and was recorded with a digital video recorder. The assessment was transcribed by a speech-language pathologist or graduate student in speech-language pathology trained in phonetic transcription.

PEEPS consists of a total of 60 words divided into two sections: a Basic Word List of 40 words for younger children (18–24 months) and an Expanded Word List of an additional 20 words for older children (24–36 months). The words were selected on the basis of two criteria: (1) age of acquisition (AOA) based on vocabulary words from the MacArthur Communicative Development Inventories [26]; (2) phonetic characteristics to elicit target English consonants across all places, voice, and manner categories of production, as well as in different syllable structures. Specifically, words were selected that are typically acquired by children between 18 and 27 months of age, as determined by Dale and Fenson [39]. The AOA for the total test words was 20.5 months (range 18–27 months) with the AOA for the Basic Word List at 19.4 months (range 18–21 months) and 22.7 months for the Expanded Word List (range 21–27 months). Thus, all but two of the total test words (96.7%) were acquired by 24 months of age. Additionally, words were selected to represent the diversity of place, manner, and voicing of English consonant production, as well as syllable structure. The Basic Word List included words to elicit production of target consonants in all seven places of English consonant articulation, all five manner classes, voiced and voiceless consonants, and simple syllable structures (CV, CVC, CVCV), along with some multisyllabic words (“peek-a-boo” and “belly button”) and a word-final nasal cluster (e.g., “hand”). The Expanded Word List builds on the Basic Word List with additional words that sample more polysyllabic words (e.g., “dinosaur”), complex syllable structures that elicit clusters in all three word positions (e.g., “truck,” “monkey,” and “drink”), and includes additional consonants in word positions that were not examined in the Basic Word List. Finally, multiple exemplars of the different phonetic characteristics were included across a number of words in order to check consistency and variability of children's early phonological skills.

The children were seated at a table or on the floor. A wireless microphone was worn in an adapted shirt and a video camera was placed on a tripod with full-face view of the child. An assistant was available to reposition the camera to maintain full-face view. A brief 5-minute warm-up activity with novel toys was available if the children did not immediately engage with toy manipulatives. The words were elicited in any order through interaction with toy manipulatives. The manipulatives were placed in a soft sack that was used to playfully engage the child in pulling the items out of the bag and naming each one as it was pulled out. Only a few items were placed in the sack at a time and the child was asked to pull out one toy at a time. A hierarchy of elicitation strategies was employed to facilitate naming of the target words. At the first level, *spontaneous elicitation* was attempted by simply asking “What is that?” If the child did not label the toy, the child was prompted by the clinician and then asked them again “What is this?” If the child still did not spontaneously label the item, then *sentence completion* was used; for example, “The baby is sleepy. Put the baby in the…(bed).” The third level of elicitation involved *modeling* the word by saying “It's a fish!” Look at this pretty fish. Would you like this? What do you want?” If the child gave a different label for a target word, then the clinician recast it with a model and then asked a question to elicit the word. For example, “That's right, you can call this a “baby,” but I sometimes call it a “doll.” What do I call it?” If the child still did not label the item after the model, then the toy was put back in the sack and the child had the opportunity to label the toy later. Following the assessment, the children's responses were phonetically transcribed using the International Phonetic Alphabet [40].

### 2.3. Measures of Phonological Development

Phonologic components assessed included phonetic inventory (word initial, medial, and final), including segmental characteristics of *place* (labial, alveolar, and velar), *voice*, and *manner* of consonant production, syllable structure (production of two and three syllable words, initial consonant clusters); accuracy (Percentage of Consonants Correct: PCC; [41, 42]); error patterns (presence of common phonological processes of substitution or deletion; compensatory error use for children with CLP); nasal emission (percent of words with nasal emission, for CLP children).

### 2.4. Procedural Fidelity and Reliability

The procedural fidelity of the PEEPS administration was assessed through coding of 50% of the assessment session for each child. Videotapes of the testing sessions were reviewed and coded by a graduate student in speech-language pathology not associated with the assessments of the children.  Table 1 displays the average percent agreement for the children with CLP and without clefts for the five major components of the administration. All components for both groups achieved a reliability score of ≥91% agreement.

 Transcription reliability was performed on 25% or more of each child's assessment by a speech-language pathologist or graduate student familiar with the phonetic transcription of young children with phonological disorders. In order to be considered an agreement, the consonant needed to be transcribed identically for place, manner, and voicing. Interjudge reliability ranged from 78% to 94% (mean 85%) for children with CLP and 90%–96% (mean 93%) for the children without clefts. A weighted *κ* was calculated to examine transcription agreement based on the frequency of occurrence of different manner classes. A *κ* of  .76 for the children with CLP and  .85 for the noncleft children was obtained indicating excellent agreement for both groups [43]. However, since many of the disagreements that did occur involved compensatory articulation errors, these disagreements were examined further. Forty-five percent (32/72) of the disagreements included compensatory errors of glottal stops (i.e., /k in duck), pharyngeal fricatives (i.e., /in shoe), and posterior nasal fricatives (i.e., /s in sock). All words with disagreements were retranscribed by two senior investigators to reach consensus.

## 3. Results and Discussion

### 3.1. Phonetic Inventory

Table 2 shows the mean, standard deviation, *t*-test, probability, and effect size for three measures describing the children's phonetic inventory. The total number of consonants correct on the PEEPS showed significantly fewer correct consonants for the children with CLP when compared to the NC children (*t* = 9.88, *P* ≤ 0.0001, *d* = 2.91). When initial and medial/final consonants were examined separately, both variables showed significantly fewer consonants than the NC group (*t* = 8.66, *P* ≤ 0.0001, *d* = 2.65; *t* = 11.37, *P* ≤ 0.0001, *d* = 3.47). Effect sizes (Cohen's *d* [44]) were calculated using G*Power (version 3.0.10; http://www.psycho.uni-duesseldorf.de/aap/projects/gpower). Values ranged from −4.72 to 4.72 for the statistically significant comparisons, indicating large effect sizes (see Table 2).

**Figure 1:**
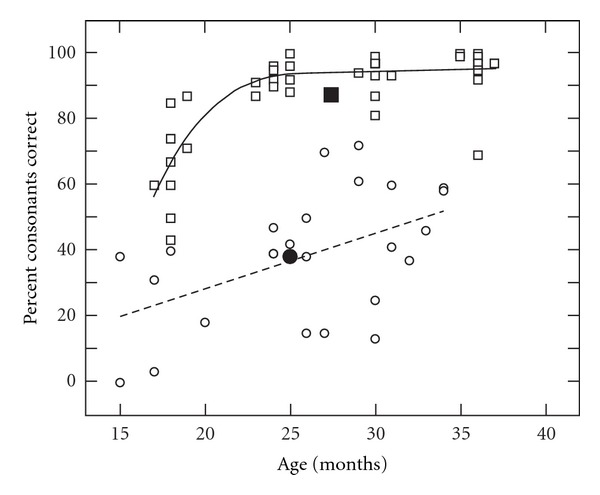
The Percentage of Consonants Correct (PCC) used on the PEEPS for children with CLP (circles) and NC children (squares) across the ages of 18 to 36 months. The linear (CLP: dotted line) and curvilinear (NC: solid line) polynomials and means (filled symbols) are displayed for the groups.

**Figure 2:**
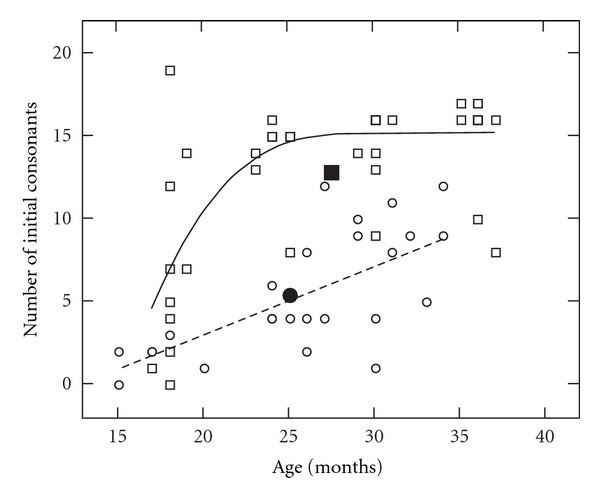
Number of initial consonants in the phonetic inventories of the children with and without CLP across age determined from the PEEPS. The number of correct initial consonants used on the PEEPS for children with CLP (circles) and NC children (squares) across the ages of 18 to 36 months. The linear (CLP: dotted line) and curvilinear (NC: solid line) polynomials and means (filled symbols) are displayed for the groups.

 The growth in acquisition of initial and medial/final consonants is shown in Figures 1 and 2.  Figure 1 shows the number of correct initial consonants used on the PEEPS for children with CLP (circles) and NC children (squares) across the ages of 18 to 36 months. The linear (CLP: dotted line) and curvilinear (NC: solid line) polynomials and means (filled symbols) are displayed for the groups. For the NC children, there is substantial growth in consonant production between 18 and 24 months of age. This corresponds with the rapid growth that typically occurs as children move from the unanalyzed whole-word stage to a rule-governed stage of phonological acquisition. The children with CLP showed a gradual improvement in consonant production over time, as indicated by the slope of the linear regression.  Figure 2 illustrates the number of correct medial/final consonants on the PEEPS for children with CLP (circles) and NC children (squares) across the 18 to 36 month ages. The children with CLP produced fewer medial/final consonants correct than initial consonants (shown in Figure 1) whereas the NC children produced similar numbers of medial/final and initial consonants correct. The pattern of variability in early sound production for NC children at 18–24 months of age appears for medial/final consonants whereas the children with CLP show gradual increase in correct sound production through 36 months of age.

 The presence or absence of three places of articulation features (labial, alveolar, velar) was examined for both groups. The children with CLP showed the presence of fewer place contrasts than the NC children. Particularly, the children with CLP produced fewer alveolar-velar contrasts (*χ* ^2^ = 11.28, *P* = 0.001), labial-velar contrasts (*χ* ^2^ = 15.9, *P* = 0.001), and presence of all three contrasts (*χ* ^2^ = 18.8, *P* = 0.001) than did the children with NC. The groups did not differ in the presence of labial-alveolar contrasts (*χ* ^2^ = 3.42, *P* = 0.064). 

### 3.2. Syllable Structure

 The production of consonants correct in two and three syllable words on the PEEPS was examined for both groups and showed no significant differences between the groups (*χ* ^2^ = 2.25, *P* = 0.13; *χ* ^2^ = 0.05, *P* = 0.81). The consonants correct within initial consonant clusters, however, showed significantly fewer correct productions for the children with CLP than the NC children (*t* = 4.58, *P* ≤ 0.0001, *d* = 1.38). Final consonant clusters could not be compared because so few children in either group produced them.

### 3.3. Accuracy

 The Percent Consonants Correct (PCC; [41]) was calculated for the total PEEPS sample and separately by manner class (see Table 2). The mean total PCC for the children with CLP was 34.9% compared to the NC children with 86.5% (*t* = 9.83, *P* ≤ 0.0001, *d* = 2.89). When the manner class PCCs were compared, nasals, stops, fricatives, affricates, and liquids showed significantly poorer scores for the children with CLP than the NC children. The glides did not show a difference between the groups. The effect sizes for all comparisons were high, ranging from a *d* of 1.67 to 4.72.

**Figure 3:**
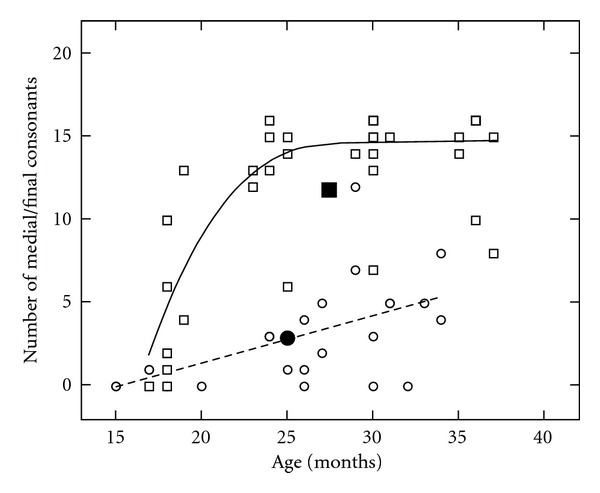
Number of medial and final consonants in the phonetic inventory of children with CLP (circles) and NC children (squares) across the ages of 18 to 36 months. The linear (CLP: dotted line) and curvilinear (NC: solid line) polynomials and means (filled symbols) are displayed for the groups.

Figure 3 summarizes PCC for the total PEEPS sample for children with CLP (circles) and NC children (squares) from 18 to 36 months of age. The growth in PCC was greatest between 18 and 24 months of age for the NC children. In contrast, the children with CLP showed slow, gradual acquisition of consonant production over time.

### 3.4. Error Analysis

 The percent of words with sound errors on the PEEPS indicated a large difference between the groups, with the children with CLP significantly higher (Mean = 76.3%, SD = 17) than the NC children (Mean = 10.7%, SD 9.8; *t* = −15.69, *P* ≤ 0.0001, *d* = −4.72). An analysis of the error types indicated that children with CLP used more substitutions and omissions than did the NC children (*t* = −6.44, *P* ≤ 0.0001, *d* = −2.0; *t* = −4.54, *P* ≤ 0.0001, *d* = − 1.42).

**Figure 4:**
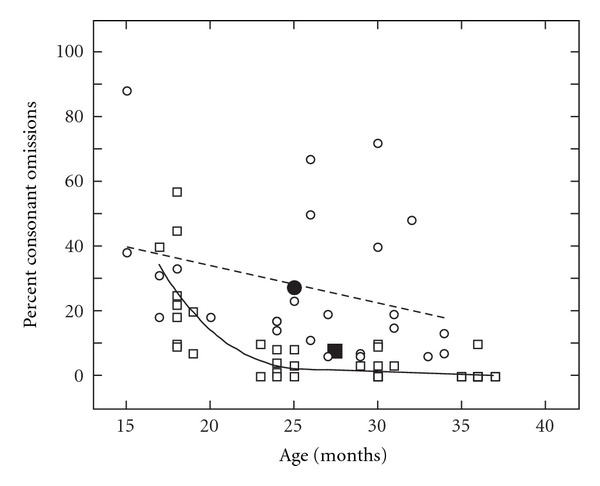
Percent of consonant omissions determined from the PEEPS for the children with CLP (circles) and NC children (squares) across the ages of 18 to 36 months. The linear (CLP: dotted line) and curvilinear (NC: solid line) polynomials and means (filled symbols) are displayed for the groups.

**Figure 5:**
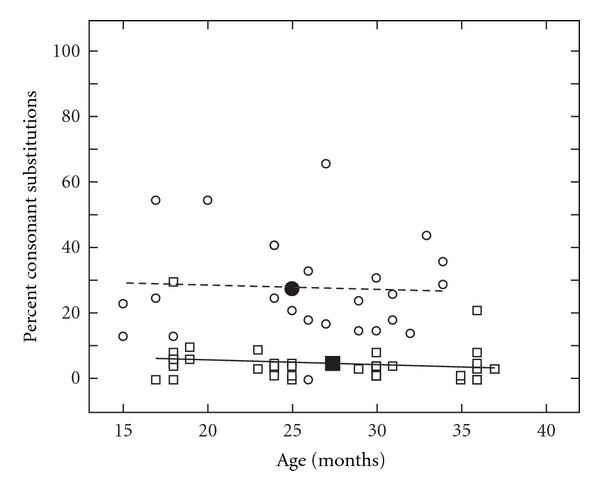
Percent of consonant substitutions determined from the PEEPS for the children with CLP (circles) and NC children (squares) across the ages of 18 to 36 months. The linear (CLP: dotted line) and curvilinear (NC: solid line) polynomials and means (filled symbols) are displayed for the groups.

 Figures 4 and 5 represent the percent of consonant omissions and substitutions on the PEEPS for children with CLP (circles) and NC children (squares) across ages from 15 to 37 months of age. The NC children showed a rapid decrease in their consonant omission and substitution patterns from 18 to 24 months of age whereas the children with CLP demonstrated a gradual reduction in omission and substitution patterns across the 18–36 month range.

### 3.5. Compensatory Errors

 The children with CLP used a mean percent of compensatory errors on the PEEPS of 7.0, SD 6.8; the NC group used none. Fifteen of the 26 children with CLP used compensatory errors at least twice during the sample. Thirteen of the 15 children had fewer than 10 (3–13% of the words in their sample) compensatory errors in the sample. The remaining two children had 13 and 14 errors, respectively (18% of the words in their sample). Glottal stops were the predominant compensatory error used, with pharyngeal fricative the second most used error pattern. Posterior nasal fricatives were used 1-2 times by three children.

### 3.6. Nasal Emission

Nasal emission was coded on consonant production of the PEEPS. Three of the 26 children with CLP used nasal emission on more than 2 occurrences. The three children used nasal emission on an average of eight words on the PEEPS. Additionally, these three children also used 1-2 occurrences of posterior nasal fricative substitutions. No direct measurements of velopharyngeal function were conducted on these young children.

## 4. Conclusions and Discussion

 The PEEPS protocol provided a systematic and developmentally appropriate means of assessing differences between a group of children with and without early speech delays. PEEPS provided a profile of early phonological development that captured consonant inventory, syllable structure, accuracy, and error analysis from 18 to 36 months of age. From the results from this study, PEEPS revealed significant differences between typical phonological development and delays across all four major parameters in comparison to the children with CLP. Additionally, the rate of growth revealed that children with CLP were slower than the NC children in all phonological measures. Finally, qualitative differences in addition to quantitative differences were noted with regard to compensatory errors and nasal emission.

 In conclusion, the following observations can be summarized from these results.

– The typical profiles at 18 months reflect what is known about normal phonological acquisition with regard to individual variability and a whole-word strategy.
– The shift to a rule-based strategy at 24 months for typically developing toddlers was observed with variability across children being largely extinguished at that age.
– The CLP children mirrored normal development at a slower pace; in general, reflecting the 18-month NC children at 30 months, which was a full 12 months later.

### 4.1. Clinical Implications

This first examination of PEEPS [1] suggests that it provides an instrument to assess reliably the phonetic and phonological development of typical and atypical children between the ages of 18 and 36 months. Additionally, the data from the children with CLP could be used to design appropriate intervention strategies. For example, if the phonological profile of a child with CLP reflects a whole-word stage of lexical and phonological acquisition, implementation of a broad-based intervention strategy directed to an emerging sound system might be indicated. This could include a parent-implemented naturalistic language approach rather than a more structured, rule-based phonological approach. PEEPS, therefore, provides a thorough and developmentally appropriate method for assessing the phonological skills of toddlers that can allow for early intervention for phonological disorders. 

### 4.2. Future Research Directions

These results represent a preliminary examination of PEEPS and additional studies with larger samples of typically and atypically developing children are needed. In addition, future studies are needed that compare PEEPS to a reference standard, such as a language sample, to evaluate test specificity and sensitivity. 

## Figures and Tables

**Table 1:** Percent agreement for procedural components of the PEEPS assessments for the children with CLP and NC children.

Procedure	CLP	NC
Correctly presents stimulus	98%	94%
Follows correct prompt sequence	96%	94%
Responds to child's questions/requests	92%	93%
Praises for engagement rather than correct response	91%	92%

**Table 2:** Mean and standard deviation for PEEPS measures for children with and without cleft palate and statistical comparisons.

Measure	NC		Cleft		*t*-test	*P*	d*
	Mean	SD*	Mean	SD*			
Phonetic Inventory							
No. Consonants	65.9	11.5	25.8	15.7	9.88	≤0.001	2.91
No. Initial Cons	14.6	2.5	6.9	3.3	8.66	≤0.001	2.65
No. Medial/Final Cons	14.0	2.7	4.0	3.0	11.37	≤0.001	3.47
Syllable Structure							
Initial Clusters	1.3	0.7	0.4	0.7	4.58	≤0.001	1.38
Accuracy							
PCC-Total	86.5	15.1	34.9	20.2	9.83	≤0.001	2.89
PCC-Nasals	97.9	5.2	66.9	25.7	5.05	≤0.001	1.67
PCC-Glides	78.1	42.0	69.2	48.0	0.58	0.567	0.19
PCC-Stops	96.0	6.0	47.3	25.2	8.05	≤0.001	2.65
PCC-Fricative	94.5	8.0	29.6	11.8	14.76	≤0.001	4.72
PCC-Affricates	79.7	33.3	11.8	28.1	7.54	≤0.001	2.20
PCC-Liquids	84.3	16.1	24.1	18.8	11.44	≤0.001	3.43
Error Analysis							
% Word Errors	10.7	9.8	76.3	17.0	−15.69	≤0.001	−4.72
% Substitutions	3.5	3.7	23.9	13.9	−6.44	≤0.001	−2.00
% Omissions	2.3	3.5	16.6	13.9	−4.54	≤0.001	−1.42

*Note. SD: Standard deviation, *d*: Cohen's.
